# Defining minimum image quality criteria for common diagnostic point‐of‐care ultrasound images: A position statement of the Society of Hospital Medicine

**DOI:** 10.1002/jhm.70156

**Published:** 2025-09-22

**Authors:** James Anstey, Ajay Bhasin, Ricardo Franco‐Sadud, Anna Maw, Benji K. Mathews, Ria Dancel, David M. Tierney, Elizabeth K. Haro, Joel Cho, Christopher Schott, Brandon Boesch, Gigi Y. Liu, Kreegan Reierson, Trevor P. Jensen, Robert Nathanson, Carolina Candotti, Gordon Johnson, Tanping Wong, Gerard Salame, Benjamin Galen, Gregory Mints, Renee Dversdal, Jason Williams, Linda Kurian, Charles M. LoPresti, Jeremy S. Boyd, Ernest Fischer, Summer L. Kaplan, Amer M. Johri, Luyao Shen, Rob Arntfield, Mangala Narasimhan, Paul Mayo, Nilam J. Soni

**Affiliations:** ^1^ Division of Hospital Medicine Oregon Health and Science University Portland Oregon USA; ^2^ Division of Hospital Medicine University of Michigan Ann Arbor Michigan USA; ^3^ Naples Comprehensive Health System University of Central Florida Naples Florida USA; ^4^ Division of Hospital Medicine University of Colorado Denver Denver Colorado USA; ^5^ Department of Hospital Medicine Health Partners Bloomington Minnesota USA; ^6^ Department of Medicine University of Minnesota Medical School Minneapolis MN USA; ^7^ Division of Hospital Medicine University of North Carolina Chapel Hill North Carolina USA; ^8^ Division of General Pediatrics and Adolescent Medicine University of North Carolina Chapel Hill North Carolina USA; ^9^ Department of Medical Education Abbott Northwestern Hospital Minneapolis Minnesota USA; ^10^ Research Service South Texas Veterans Health Care System San Antonio Texas USA; ^11^ Division of Pulmonary Diseases & Critical Care Medicine University of Texas Health San Antonio San Antonio Texas USA; ^12^ Department of Hospital Medicine Kaiser Permanente Medical Center San Francisco California USA; ^13^ Critical Care Service Veterans Affairs Pittsburgh Health Care Systems Pennsylvania USA; ^14^ Departments of Critical Care Medicine and Emergency Medicine University of Pittsburgh Pennsylvania USA; ^15^ Hospital Medicine Program, Cottage Health Santa Barbara California USA; ^16^ Division of Hospital Medicine, Department of Medicine Johns Hopkins School of Medicine Baltimore Maryland USA; ^17^ Department of Medicine, Division of Hospital Medicine University of California San Francisco San Francisco California USA; ^18^ Section of Hospital Medicine South Texas Veterans Health Care System San Antonio Texas USA; ^19^ Department of Medicine, Division of Hospital Medicine University of Texas Health San Antonio San Antonio Texas USA; ^20^ Division of Hospital Medicine University of California Davis Davis California USA; ^21^ Division of Hospital Medicine Legacy Healthcare System Portland Oregon USA; ^22^ Division of Hospital Medicine Weill Cornell Medicine New York NY USA; ^23^ Saint Joseph Hospital Denver Colorado USA; ^24^ Department of Medicine, Division of Hospital Medicine Albert Einstein College of Medicine and Montefiore Medical Center New York New York USA; ^25^ Section of Hospital Medicine Atlanta Veterans Affairs Medical Center Atlanta Georgia USA; ^26^ Division of Hospital Medicine Emory School of Medicine Atlanta Georgia USA; ^27^ Division of Hospital Medicine Zucker School of Medicine at Hofstra Northwell New Hyde Park New York USA; ^28^ Hospital Medicine Service University Hospitals Cleveland Ohio USA; ^29^ Department of Medicine Case Western Reserve University School of Medicine Cleveland USA; ^30^ Department of Emergency Medicine Vanderbilt University Nashville Tennessee USA; ^31^ Department of Emergency Medicine Veterans Affairs Tennessee Valley Healthcare System Nashville Tennessee USA; ^32^ Department of Medicine MedStar Georgetown University Hospital Washington DC USA; ^33^ Department of Radiology, Children's Hospital of Philadelphia Perelman School of Medicine University of Pennsylvania Philadelphia Pennsylvania USA; ^34^ Department of Medicine, Division of Cardiology Queen's University Kingston Canada; ^35^ Department of Radiology Stanford University School of Medicine Stanford California USA; ^36^ Division of Critical Care Western University London Ontario Canada; ^37^ Division of Pulmonary, Critical Care, and Sleep Medicine, Northwell Health LIJ/NSUH Medical Center Donald and Barbara Zucker School of Medicine at Hofstra/Northwell Hempstead New York USA

## Abstract

**Background:**

Point‐of‐care ultrasound (POCUS) use continues to expand across multiple clinical subspecialties, and the need for standardization of training and quality assurance has become increasingly important. Despite the need for training, there are currently no widely accepted multispecialty criteria to define an acceptable quality POCUS image for common POCUS applications used by clinicians. Without such criteria, discrepancies in rating POCUS image quality occur, leading to inconsistencies in training and quality assurance, which can ultimately compromise patient care and safety.

**Methods:**

To address this gap, the Society of Hospital Medicine (SHM) Point‐of‐care Ultrasound Task Force convened an expert panel of 32 national POCUS experts trained in hospital medicine (*n* = 24), critical care (*n* = 4), emergency medicine (*n* = 3), radiology (*n* = 2), and cardiology (*n* = 1) and employed a modified‐Delphi approach to develop minimum image quality criteria for five common POCUS applications: heart, lungs, abdomen, lower extremity veins, and skin/soft tissues.

**Results:**

After three rounds of voting and group discussion, the panel achieved consensus on a comprehensive list of 215 items to define standard image quality criteria in five different body systems.

**Conclusions:**

These POCUS image quality criteria offer a structured, consensus‐based framework for evaluating POCUS images and establish a minimum standard for defining an acceptable quality image. Use of these criteria can improve inter‐rater reliability and advance standardization of POCUS imaging, which affects training, quality assurance, and credentialing/privileging practices.

## INTRODUCTION

In recent years, point‐of‐care ultrasound (POCUS) has transformed the bedside evaluation of patients. This versatile tool provides real‐time diagnostic information across a wide range of applications, which enhances clinical decision‐making, improves diagnostic accuracy, and shortens the time to correct diagnosis.^1^, ^2^, ^3^, ^4^, ^5^ Adoption of POCUS continues to spread rapidly, and integration of POCUS across specialties has highlighted the need for standardized approaches to training, assessment of competency, and quality assurance.

Early guidelines in POCUS recommended learners perform a specific number of scans to establish adequate skills in image acquisition.^6^, ^7^ Since then, there has been a shift from numerical cutoffs to competency‐based education in which evaluation of image quality is a more effective method of assessing competence in image acquisition.^8^ As more trainees and clinicians are seeking POCUS training worldwide, actively supervising image acquisition of all learners at the bedside is not feasible, and a commonly accepted surrogate for building adequate image acquisition skills is the development of an image portfolio with asynchronous review by POCUS experts.^9^, ^10^, ^11^ Given that mastery of image acquisition is the most challenging and time‐consuming part of achieving competency in POCUS,^12^ the image portfolio has become an important pillar of POCUS training. In addition to evaluating image acquisition skills, ongoing ultrasound image review is central to quality assurance of most POCUS programs.^13^, ^14^

Despite the importance of assessing image quality for portfolio development in POCUS training and quality assurance, there is little formal guidance on what constitutes an adequate quality ultrasound image, leaving learners and educators to rely on individual judgment of POCUS image quality. Several societies have published guidelines on POCUS use, but specific image quality criteria are generally not addressed.^3^, ^7^, ^15^, ^16^, ^17^, ^18^ Image quality scoring protocols have been developed for reviewing images of specific exam types, such as cardiac images, but none specify the image requirements for defining an acceptable quality image across common POCUS applications.^19^, ^20^, ^21^, ^22^, ^23^ Due to the absence of clear and consistent image quality standards, a moderate level of agreement has been demonstrated when inter‐rater reliability was assessed among POCUS experts,^24^, ^25^, ^26^, ^27^, ^28^, ^29^, ^30^ and intra‐ and interinstitutional differences in standards for POCUS training and quality assurance are common.

To address this gap in POCUS imaging, we recruited a large, multispecialty group of POCUS experts to establish standardized image quality criteria for common POCUS applications. A systematic and iterative methodology was used to synthesize the collective expertise of physicians from diverse backgrounds to achieve consensus on image quality criteria. By establishing these criteria, we aim to support standardization of POCUS imaging across various medical specialties and promote safe and effective POCUS use.

## METHODS

We conducted a prospective observational study using consensus‐based methods in two phases. The Institutional Review Board of the University of Texas Health Science Center San Antonio reviewed and deemed this study to be exempt from review (Protocol Number: 20230415EX).

The initial POCUS image quality criteria were developed in 2019 through a series of group discussions of hospitalist POCUS experts who served on the Society of Hospital Medicine Point‐of‐care Ultrasound Certificate of Completion Steering Committee (*n* = 10). Five diagnostic applications (heart, lungs, abdomen, vascular, and skin/soft tissues) were chosen as the core applications for basic POCUS competency based on guidelines from multiple specialties. These criteria were then piloted to evaluate learners' image portfolios from 2020 to 2021, and based on image reviewer feedback, a few criteria were modified in 2021.

To gather formal consensus on POCUS image quality criteria, we convened an expert panel of 32 national POCUS faculty trained in hospital medicine (*n* = 24), critical care (*n* = 4), emergency medicine (*n* = 3), radiology (*n* = 2), and cardiology (*n *= 1). POCUS experts were defined as having ≥3 years of experience as an attending physician grading the quality of POCUS images, teaching experience from national or international POCUS courses, and active participation in SHM or other national society POCUS activities (course faculty, special interest groups, and other POCUS leadership roles). All experts completed three rounds of asynchronous online voting, participated in ≥80% of group conference calls, contributed to drafting and finalizing the manuscript, and disclosed any conflicts of interest at three time points (start and finish of the project and prior to manuscript publication).

The initial image quality criteria checklist included five different body systems with POCUS applications (heart, lung, abdomen, lower extremity veins for deep venous thrombosis [DVT], and skin/soft tissues) with a total of 229 unique checklist items. The checklist included sections for probe type, exam preset, orientation, depth, gain, imaging plane, anatomical structures, and required findings for important pathologies. The checklist items were entered into an internet‐based electronic data collection instrument (Research Electronic Data Capture, REDCap). Expert panel members voted to “agree” versus “disagree” to include each item as a minimum requirement to define an adequate quality image. Experts were encouraged to write comments but required to provide feedback in free‐text boxes if they disagreed with the inclusion of an item.

We conducted three rounds of electronic voting followed by group discussion between August 2023 and October 2023. We used a modified Delphi approach to assess the level of agreement among experts, and consensus was defined as ≥80% of experts agreeing to include an item. Items achieving <80% consensus for inclusion were discussed, revised, and re‐considered during Round 2 of voting. Additionally, we discussed items with >80% agreement if important comments were made during voting (e.g., adjusting depth criteria for pleural effusions). Based on the group discussion, minor changes to wording of some items were made. The final checklist was reviewed during Round 3 of voting. Two items (ascites and cellulitis) were removed from the initial checklist after Round 1 voting. Our goal was to establish minimal image quality criteria for standard views, and pathological findings that were captured well with standard views were not voted on separately, except for a few common lung pathologies that may require alteration of the standard imaging technique, such as pleural effusions and lung consolidation. All expert panel members reviewed the final image quality criteria checklist before submission for publication.

## RESULTS

Thirty‐two POCUS experts representing all regions of the United States participated and completed all three rounds of voting (Table 1). Most experts were male and specialized in internal medicine. More than half of the experts had been practicing medicine, using and teaching ultrasound for ≥10 years. Most faculty (84%) held local or national POCUS leadership positions and had ≥10 ultrasound‐related peer‐reviewed journal publications. Three faculty abstained from voting on portions of the checklist where they lacked appropriate expertise (two radiologists and one cardiologist refrained from voting on cardiac and noncardiac images, respectively).

**Table 1:** Characteristics of the point‐of‐care ultrasound expert panel (*n* = 32).

Characteristics	*N* (%)
Primary specialty	
Internal medicine/hospital medicine	24 (75%)
Critical care	4 (13%)
Emergency medicine	1 (3%)
Radiology	2 (6%)
Cardiology	1 (3%)
Gender	
Female	10 (31%)
Geographic region	
Northeast & Canada (CT, DE, MA, ME, NH, NJ, NY, PA, RI, VT)	10 (31%)
South (AL, AR, FL, GA, KY, LA, MD, MS, NC, OK, SC, TN, TX, VA, WV)	8 (25%)
Midwest (IN, IA, IL, KS, MI, MN, MO, ND, NE, OH, SD, WI)	4 (13%)
West (AK, AZ, CA, CO, HI, ID, MT, NM, NV, OR, UT, WA, WY)	10 (31%)
Completed ultrasound fellowship	
Yes	5 (16%)
Years practicing medicine since terminal residency/fellowship	
0–10 years	5 (16%)
11–15 years	15 (46%)
>15 years	12 (38%)
Years using ultrasound in clinical practice	
0–10 years	12 (38%)
11–15 years	8 (25%)
≥16 years	12 (38%)
Years teaching ultrasound to other clinicians	
0–10 years	13 (41%)
11–15 years	11 (34%)
≥16 years	8 (25%)
Years assessing ultrasound skills of other clinicians	
0–10 years	17 (53%)
11–15 years	9 (28%)
≥16 years	6 (19%)
Ultrasound‐related peer‐reviewed publications	
0–10	11 (34%)
11–20	13 (41%)
≥20	8 (25%)
Leadership positions related to point‐of‐care ultrasound	
Yes	27 (84%)

**Figure 1:**
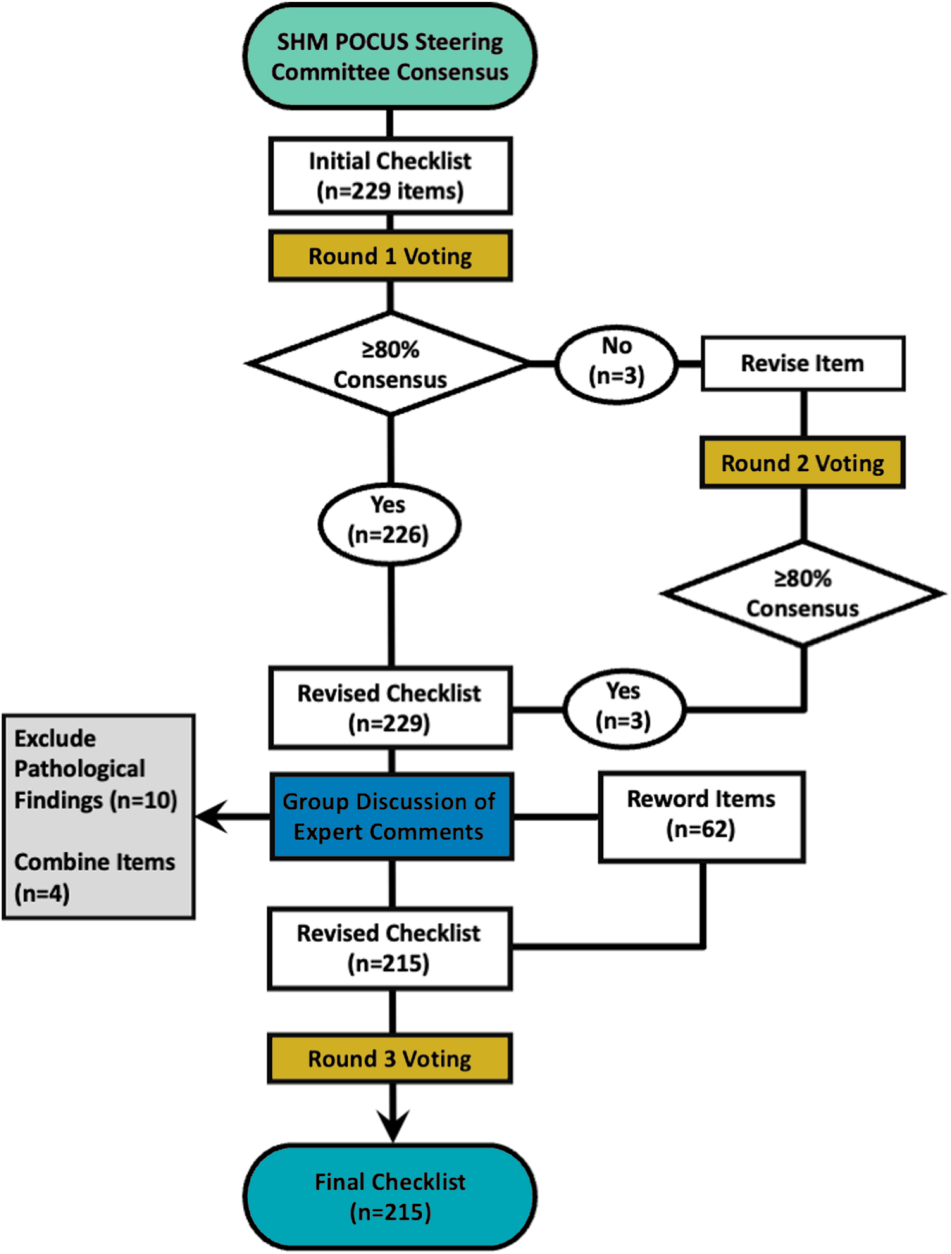
Development of consensus‐based image quality criteria for point‐of‐care ultrasound images using a modified Delphi approach.

A total of 229 checklist items of POCUS image quality in five body systems (heart, lungs, abdomen, lower extremity veins, and skin/soft tissues) were presented to the expert panel for three rounds of voting to achieve consensus (Figure 1). After the first round of voting, 226 of 229 checklist items achieved consensus. Three items that did not meet ≥80% consensus were related to lung ultrasound imaging techniques and minimum criteria to identify common lung pathologies. These three items were revised and underwent an additional round of voting, and consensus was achieved after Round 2 of voting. Before Round 3 of voting, a series of online meetings were conducted to discuss several items (*n* = 62) that met ≥80% consensus but had impactful comments that justified rewording the items. For these 62 items, the wording was either clarified to decrease ambiguity or relaxed to establish more achievable minimum image quality criteria. Additionally, 10 items for visualizing pathologic findings (ascites and cellulitis) were removed because standard imaging techniques and views were adequate for their evaluation, and four items were combined to reduce redundancy. The revised checklist was reviewed during Round 3 of voting, and all items achieved consensus without any major comments. The final checklist included 215 items across five body systems (Appendix S1): heart (Table 2), lungs/pleura (Table 3), abdomen (Table 4), lower extremity veins for DVT evaluation (Table 5), and skin/soft tissues (Table 6).

**Table 2:** Cardiac ultrasound image quality criteria.

	Vote
**Cardiac images**	
Probe: Phased array	100%
Preset: Cardiac	100%
Orientation marker: Upper right (*may vary based on institution or clinical specialty)	97%^a^
**Parasternal long‐axis view**	
Image orientation: base of the heart (AV and LA) on screen right	100%
Depth: Must include descending thoracic aorta (typically 13–16 cm); should not include excessive depth beyond the descending thoracic aorta	97%
Gain: Balanced gray‐scale image in near and far field with blood appearing anechoic in all chambers	88%^a^
Imaging plane: Ultrasound beam aligned over the center of long axis of LV with LV cavity at fullest diameter	88%^a^
Structures:	–
AV visible and slightly right of midline	91%^a^
MV visible and approximately centered in the image	97%
RVOT is visible	97%
LV is visible (*Apex is generally not visible)	91%^a^
LA is visible	100%
Aortic Root is visible	100%
**Parasternal short‐axis view (aortic valve level)**	
Image orientation: RA on screen left. RVOT on screen right. RV on top. LA on bottom. AV in center	88%^a^
Depth: Includes inferior wall of the LA without excessive depth	100%
Gain: Balanced gray‐scale image in near and far fields	97%
Imaging plane: Aligned over AV with AV leaflets, RA, RV, and LA seen	97%
Structures:	–
Right coronary cusp (CC), non‐CC, left CC of the AV should be seen (zoom if needed)	88%
LA, Interatrial Septum, RA, TV, and RV should be visible	97%
**Parasternal short‐axis view (mitral valve level)**	
Image orientation: LV on screen right. RV on left or center of screen.	97%^a^
Depth: Include the inferior wall of the LV avoiding excessive depth.	100%
Gain: balanced gray‐scale image in near and far field	97%
Imaging plane: Aligned over MV. Avoid oblique imaging planes that make the LV look oval or oblong.	100%
Structures:	–
Anterior and posterior leaflets of the MV should be seen	97%
Septum and RV visible (entire RV free wall may not be seen)	97%^a^
Endocardium of all LV walls (anterior, septal, inferior, lateral) are visible.	97%
**Parasternal short‐axis view (mid‐papillary level)**	
Image orientation: LV on screen right. RV is on the left or center of screen.	94%^a^
Depth: Sufficient to include inferior wall of LV but avoiding excessive depth.	100%
Gain: Balanced gray‐scale image in near and far field	97%
Imaging plane: Beam should be aligned over the papillary muscles of LV in a short‐axis mid‐ventricular view. MV is not seen. Avoid oblique imaging planes that make LV look oval or oblong.	100%
Structures:	–
Both papillary muscles are visible and symmetric	97%
Septum and RV visible	97%
LV endocardium of all walls visible.	97%
**Parasternal short‐axis view (apical level)**	
Image orientation: LV should be in the center of screen.	100%
Depth: Sufficient to include the inferior wall of the LV but avoiding excessive depth.	94%^a^
Gain: Balanced gray‐scale image in near and far field	97%
Imaging plane: Beam should be aligned over apex. The papillary muscles should not be seen. Avoid oblique imaging planes that make LV look oval or oblong.	100%
Structures:	–
LV endocardium of all walls visible.	100%
**Apical four‐chamber view**	
Image orientation: RA and RV on screen left, LA and LV on screen right.	100%
Depth: Sufficient to include entirety of both atria	100%
Gain: Balanced gray‐scale image in near and far field	97%
Imaging plane:	–
Apex centered on the screen with interventricular septum vertical. Ventricles should be elongated. Normally, LV is oval and RV is triangular.	97%
Lateral walls of both the RV and LV should be visible (entire RV free wall may not be seen).	88%^a^
Posterior coronary sinus should not be visible (under tilting/tilted too posteriorly).	97%
Aortic root is not visible in an apical 4‐chamber view (over tilting/tilted too anteriorly).	97%^a^
Structures:	–
TV well visualized including valve tips indicating RV is at the greatest diameter	97%
MV well visualized including valve tips indicating that the LV is at its greatest diameter	100%
LV visible	100%
LA visible	100%
RV visible (entire RV free wall may not be seen).	88%^a^
RA visible	97%
**Subcostal four‐chamber view**	
Image orientation: Base of heart (LA and RA) is on screen left and ventricles are on screen right.	94%^a^
Depth: Sufficient to include inferolateral wall of LA in the far field	100%
Gain: Balanced gray‐scale image in near and far field	97%
Imaging plane:	
All four chambers should be clearly seen	100%
Aortic valve and aortic root not seen	97%
Ventricles elongated; normally, LV is oval and RV is triangular shape (apex is generally not seen)	94%^a^
Structures:	–
TV visible	100%
MV visible	100%
LV visible	100%
RV visible	100%
RA visible	100%
LA visible	100%
Liver and diaphragm visible	83%
**Inferior vena cava (longitudinal view)**	
Image Orientation: RA/diaphragm on the indicator side of screen; screen indicator cephalad	93%
Depth: Deep enough to view the IVC in center of screen	100%
Gain: Balanced gray‐scale image in near and far field	97%
Imaging plane:	–
Beam centered over IVC in longitudinal plane, avoiding oblique views. Anterior and posterior walls of the IVC parallel, appearing as thin, hyperechoic lines across majority of the screen.	94%^a^
Should visualize IVC connecting with RA	100%
Structures:	–
Liver and diaphragm visible	100%
IVC in long axis is visible	100%
Hepatic vein is visible (may not be seen in all patients)	94%^a^

Abbreviations: AV, aortic valve; IVC, inferior vena cava; LA, left atrium; LV, left ventricle; MV, mitral valve; RA, right atrium; RV, right ventricle; RVOT, right ventricular outflow track; TV, tricuspid valve.

^a^ Items that achieved >80% consensus but wording was adjusted per expert feedback before final round of voting.

**Table 3:** Lung and pleural ultrasound image quality criteria.

	Vote
**Lung and pleural images**	
Probe: Phased‐array or curvilinear when evaluating pleura, pleural space, or parenchyma. Linear probe when evaluating pleura only.	87%^a^
Exam Preset: Abdomen or Lung	90%
Orientation Marker: Upper left‐hand corner	97%
Image Orientation: Screen left is cephalad and screen right is caudad (longitudinal plane). For corroborating views in a transverse plane, screen left is the operator's left.^b^	97%
**Normal Lung Sliding with A‐lines**	
Depth**:** Minimum depth typically 10–12 cm but should be deep enough to see greater than 2 A‐lines.	94%^c^
Gain**:** Balanced gray‐scale image in near and far fields	97%
Imaging plane: Beam perpendicular to the pleural line to bring out A‐lines. A thin, crisp pleural line is normally seen. Orientation cephalo‐caudad with rib shadows flanking both or at least one edge of the image. Slight tilting or fanning usually necessary to orient the ultrasound beam perpendicular to the pleural surface as evidenced by appearance of multiple A‐lines.	94%^c^
Structures:	–
Pleural line visible	100%
A‐lines (>2 should be clearly visible)	81%
Rib and rib shadow(s) visible	97%
**B‐Lines**	
Gain: Balanced gray‐scale image in near and far fields	90%
Imaging plane: Beam perpendicular to pleural line. Orientation longitudinal with rib shadows flanking one or both edges of the image	94%
Structures:	–
Pleural line visible	100%
B‐lines clearly visible and meet the definition of B‐line. (Avoid mistaking other types of comet tails for B‐lines)	90%
Rib and rib shadow(s) visible	94%
**Consolidation (or Hepatization)**	
Depth: Sufficient depth to visualize extent of consolidation or hepatization.	100%^a^
Gain: Balanced gray‐scale image in near and far fields	100%
Imaging plane: Beam perpendicular to pleural line with rib shadows flanking one or both edges of image. Indicator side of screen must be cephalad.	100%^c^
Structures:	–
Pleural line visible	90%
Consolidation of lung clearly visible	100%
Rib and rib shadow(s) visible	94%
**Pleural effusion**	
Depth: Deep enough to see spine and visualize size of pleural effusion	94%
Gain: Balanced gray‐scale image in near and far fields	100%
Imaging plane: Beam perpendicular to chest wall with diaphragm, lung parenchyma, and pleural effusion clearly visualized. Orientation longitudinal with lung parenchyma on the indicator side of screen and diaphragm on opposite side.	97%
Structures:	–
Pleural effusion visible	100%
Lung parenchyma visible, unless pleural effusion spans multiple ribs spaces.	100%
Diaphragm visible	94%
Spine visible when probe lateral	84%^a^

^a^ Items that achieved >80% consensus in Round 2 voting.

^b^ In a transverse plane, screen left is the operator's left, which is the patient's right on the anterior chest wall and patient's left on the posterior chest wall. For lung/pleural images, operators are recommended to add anatomical labels (left, right, etc.).

^c^ Items that achieved >80% consensus but wording was adjusted per expert feedback before final round of voting.

**Table 4:** Abdominal ultrasound image quality criteria.

	Vote
**Abdominal images**	
Probe: Curvilinear or phased‐array probe	97%^a^
Exam preset: abdomen (or specific preset for FAST, Aorta, Liver, etc.)	100%^a^
Screen orientation marker: Upper left‐hand corner	100%
Image orientation: In a longitudinal plane, screen left is cephalad and screen right is caudad. In a transverse plane, screen left is the operator's left, which is the patient's right on the anterior abdominal wall.	100%^a^
**Right kidney with hepatorenal recess (longitudinal view)**	
Depth: Includes entire kidney in center of the screen	94%
Gain: Balanced gray‐scale image in near and far fields	100%
Imaging plane: Beam aligned longitudinally over hilum of the kidney with both superior and inferior poles visualized by rocking or sliding during image recording. Kidney should appear oval‐shaped and not circular. Maximize kidney length with the probe oriented along the rib spaces, minimizing rib shadows.	97%^a^
Structures:	–
Renal cortex and pelvis visible (Typically, renal vessels are visualized in the pelvis but not required)	97%^a^
Superior and inferior poles of kidney visible (If both poles cannot be seen simultaneously, rock or slide probe)	97%^a^
Liver and part of hepatorenal recess visible	97%
**Right kidney (transverse view)**	
Depth: Deep enough to center kidney on the screen	100%
Gain: Balanced gray‐scale image in near and far fields	100%
Imaging plane: Beam aligned transversely through the center of the kidney with hilar vessels and collecting ducts visible.	94%
Superior and inferior poles of the kidney visualized by tilting or fanning probe.	100%
**Left kidney with splenorenal recess (longitudinal view)**	
Depth: Deep enough to include entire kidney in center of screen	97%
Gain: Balanced gray‐scale image in near and far fields	100%
Imaging plane: Beam aligned longitudinally over hilum of the kidney with both superior and inferior poles visualized by rocking or sliding during image recording. Kidney should appear oval shaped and not circular. Maximize kidney length with probe oriented along the rib spaces, minimizing rib shadows	97%^a^
Structures:	–
Renal cortex and pelvis visible (Typically, renal vessels visualized in the pelvis but not required)	97%^a^
Superior and inferior poles of kidney (If both poles cannot be seen simultaneously, rock or slide Probe)	97%
Spleen and part of splenorenal recess visible	97%^a^
**Left kidney (transverse view)**	
Depth: Deep enough to center kidney on the screen	100%
Gain: Balanced gray‐scale image in near and far fields	100%
Imaging plane: Beam aligned transversely through center of the kidney with hilar vessels and collecting ducts visible	94%
Superior and inferior poles of kidney visualized by tilting or fanning probe.	100%
**Abdominal aorta (transverse view)**	
Depth: Sufficient to visualize vertebral shadow	100%^a^
Gain: Balanced gray‐scale image in near and far fields	100%
Imaging plane**:** Beam aligned transversely and perpendicular to the walls of the aorta. The anterior and posterior walls of the circular aorta seen approximately in center of screen.	100%^a^
Structures:	–
Proximal Aorta with celiac trunk or superior mesenteric artery, IVC, and vertebral shadow visible. Diameter measured at the widest point of the proximal, mid, and distal aorta, either outer wall to outer wall or from leading edge to leading edge based on institutional protocol.	100%^a^
Mid aorta with IVC and vertebral shadow visible.	87%
Distal aorta w/transition to the common iliac arteries with vertebral shadow visible.	84%
**Abdominal aorta (longitudinal view)**	
Depth: Sufficient to visualize vertebral shadow	100%^a^
Gain: Balanced gray‐scale image in near and far fields	100%
Imaging plane: Beam aligned longitudinally over the center of aorta without any narrowing or cutting off the aorta. Two walls of the aorta seen longitudinally across screen from left to right. Beak‐shaped, oblique view of aorta avoided.	100%
Structures:	–
Proximal, mid, or distal abdominal aorta in long axis visible	97%^a^
Spine visible	97%^a^
Two branches of aorta (celiac trunk, superior mesenteric artery) visible	94%^a^
**Urinary bladder (transverse view)**	
Depth: Deep enough to see entire posterior wall of bladder. Depth should be adequate to see pelvic organs (rectum, prostate, or uterus)	97%^a^
Gain: Balanced gray‐scale image in near and far fields (Adjust far‐field gain to minimize posterior acoustic enhancement	97%^a^
Imaging plane: Beam centered transversely over bladder in center of screen. Images should capture maximum dimensions of bladder for proper assessment of volume.	100%
Structures:	–
Bladder in a transverse plane visible	100%
Pelvic organs (rectum, and prostate or uterus) should be seen in far field	97%^a^
**Urinary bladder (longitudinal view)**	
Depth: Deep enough to see posterior wall of the bladder, and rectum, prostate, or uterus.	97%^a^
Gain: Balanced gray‐scale image in near and far fields	97%^a^
Imaging plane: Beam centered longitudinally over bladder in center of screen. Images should capture maximum dimensions of the bladder for proper assessment of volume. Typically, probe is positioned over superior posterior edge of the symphysis pubis and rocked to aim the ultrasound beam toward the pelvis to capture a high‐quality view.	100%
Structures:	–
Bladder in long axis visible	100%
Pelvic organs (rectum, prostate and uterus if present) are visible in far field	97%^a^
Symphysis pubis and its shadow (screen right) visible (Shadow may not be seen if bladder is greatly distended	97%^a^

Abbreviations: FAST, focused assessment with sonography in trauma; IVC, inferior vena cava.

^a^ Items that achieved >80% consensus but wording was adjusted per expert feedback before final round of voting.

**Table 5:** Lower extremity venous ultrasound image quality criteria.

	Vote
**Lower extremity deep venous thrombosis exam images**	
Probe: Linear probe. Severe edema or high BMI may warrant selection of an alternative transducer offering increased depth at expense of lower resolution. For single‐transducer systems, choose the linear setting.	97%
Exam preset: Vascular, venous, arterial, or musculoskeletal	87%
Screen orientation marker: Upper left‐hand corner	100%
Image orientation: Screen left is the operator's left which is the patient's right on the anterior thigh and leg.^a^ Vessels should be centered on screen, avoiding excessive depth placing the vessels in the upper third of screen.	100%
**Right common femoral vein**	
Depth: Sufficient to visualize the right CFV in center of screen	100%
Gain: Balanced gray‐scale image in near and far fields^c^	100%
Imaging plane: Ultrasound beam should be transverse to target vessel and vein should appear in center of screen (avoid oblique views)^d^	100%
Structures:	–
Both right CFA (screen left) and right CFV (screen right) are visible	100%
GSV should not be visible	100%^b^
Full compressibility of right CFV demonstrated without sliding.^e^	90%^b^
**Right common femoral vein—greater saphenous vein junction**	
Depth: Sufficient to visualize CFV‐GSV in the center of the screen	100%
Gain: Balanced gray‐scale image in near and far fields^c^	100%
Imaging plane: Ultrasound beam should be transverse to target vessel and vein should appear in center of screen (avoid oblique views)^d^	100%
Structures:	–
Right CFA, CFV, and GSV all visible, with GSV extending medially and anteriorly (top right for RLE)	100%
Full compressibility of both right CFV and GSV demonstrated without sliding.^e^	100%^b^
**Right femoral vein—deep femoral vein junction**	
Depth: Sufficient to visualize the right CFV splitting into FV and DFV branches in center of screen. Note: lateral perforators are usually seen between superficial and deep femoral artery and should NOT be confused with DFV. After CFV splits into FV and DFV, DFV usually dives posteriorly and is not seen distally beyond bifurcation.	97%
Gain: Balanced gray‐scale image in near and far fields^c^	90%^b^
Imaging plane: Ultrasound beam should be transverse to target vessel and vein should appear in center of screen (avoid oblique views)^d^	100%
Structures:	–
Distal right CFV and bifurcation both visible with slight tilting or fanning of the probe	97%^b^
Right FV and DFV visible (avoid mistaking lateral perforator vein for DFV)	97%
Full compressibility of right FV and DFV, and any lateral perforators, demonstrated without sliding.^e^	91%^b^
**Right mid‐distal femoral vein**	
Depth: Sufficient to visualize the right mid‐/distal FV in center of screen	100%
Gain: Balanced gray‐scale image in near and far fields^c^	100%
Imaging plane: Ultrasound beam should be transverse to target vessel and vein should appear in center of screen (avoid oblique views)^d^	100%
Structures:	–
Right Mid‐/distal FV and superficial femoral artery visible	100%
Full compressibility of the right FV demonstrated without sliding.^e^ To improve the sensitivity of the DVT exam, compressions in ~1 cm increments down the length of the FV is recommended.	94%^b^
**Right popliteal vein**	
Depth: Sufficient to visualize right PV in center of screen and not be mistaken for superficial calf veins	100%
Gain: Balanced gray‐scale image in near and far fields^c^	100%
Imaging plane: Ultrasound beam should be transverse to target vessel and vein should appear in center of screen (avoid oblique views).^d^ Avoid capturing views too distal where the PV has bi‐ or trifurcated.	97%
Structures:	–
Right PV and PA visible	100%
Full compressibility of the right PV should be demonstrated without sliding.^e^ Avoid mistaking the anterior tibial, peroneal, or posterior tibial veins for the PV.	100%^b^
**Left common femoral vein**	
Depth: Sufficient to visualize the left CFV in center of screen	100%
Gain**:** Balanced gray‐scale image in near and far fields^c^	100%^b^
Imaging plane: Ultrasound beam should be transverse to target vessel and vein should appear in center of screen (avoid oblique views)^d^	100%
Structures:	–
Both left CFA (screen right) and left CFV (screen left) are visible	100%
GSV should not be visible	100%^b^
Full compressibility of left CFV demonstrated without sliding.^e^	94%^b^
**Left common femoral vein—greater saphenous vein junction**	
Depth: Sufficient to visualize CFV‐GSV in the center of the screen	100%
Gain: Balanced gray‐scale image in near and far fields^c^	100%^b^
Imaging plane: Ultrasound beam should be transverse to target vessel and vein should appear in center of screen (avoid oblique views)^d^	100%
Structures:	–
Left CFA, CFV, and GSV all visible, with GSV extending medially and anteriorly (top left for LLE)	100%
Full compressibility of both left CFV and GSV demonstrated without sliding.^e^	90%^b^
**Left femoral vein—deep femoral vein junction**	
Depth: Sufficient to visualize the left CFV splitting into FV and DFV branches in center of screen. Note: lateral perforators are usually seen between superficial and deep femoral artery and should NOT be confused with DFV. After CFV splits into FV and DFV, DFV usually dives posteriorly and is not seen distally beyond bifurcation.	97%
Gain: Balanced gray‐scale image in near and far fields^c^	90%^b^
Imaging plane: Ultrasound beam should be transverse to target vessel and vein should appear in center of screen (avoid oblique views)^d^	100%
Structures:	–
Distal left CFV and bifurcation both visible with slight tilting or fanning of the probe	94%^b^
Left FV and DFV visible (avoid mistaking lateral perforator vein for DFV)	97%
Full compressibility of left FV and DFV, and any lateral perforators, demonstrated without sliding.^e^	94%^b^
**Left mid‐distal femoral vein**	
Depth: Sufficient to visualize the left mid‐/distal FV in center of screen	100%
Gain: Balanced gray‐scale image in near and far fields^c^	100%
Imaging plane: Ultrasound beam should be transverse to target vessel and vein should appear in center of screen (avoid oblique views)^d^	100%
Structures:	–
Left Mid‐/distal FV and superficial femoral artery visible	100%
Full compressibility of the left FV demonstrated without sliding.^b^ To improve the sensitivity of the DVT exam, compressions in ~1 cm increments down the length of the FV is recommended.	94%^b^
**Left popliteal vein**	
Depth: Sufficient to visualize left PV in the center of screen and not be mistaken for superficial calf veins	100%^b^
Gain: Balanced gray‐scale image in near and far fields^c^	100%^b^
Imaging plane: Ultrasound beam should be transverse to target vessel and vein should appear in center of screen (avoid oblique views).^d^ Avoid capturing views too distal where the PV has bi‐ or trifurcated.	97%
Structures:	–
Left PV and PA visible	100%
Full compressibility of the left PV should be demonstrated without sliding.^e^ Avoid mistaking the anterior tibial, peroneal, or posterior tibial veins for the PV.	94%^b^

Abbreviations: CFA, common femoral artery; CFV, common femoral vein; DFV, deep femoral vein; FV, femoral vein; GSV, greater saphenous vein; LLE, left lower extremity; PV, popliteal vein; RLE, right lower extremity.

^a^ In a transverse plane, screen left is the operator's left which is the patient's right on the anterior thigh/leg and the patient's left on the posterior thigh/leg. Operators are recommended to add anatomical labels (left, right, etc.) to facilitate accurate interpretation.

^b^ Items that achieved >80% consensus but wording was adjusted per expert feedback before final round of voting.

^c^ Gain: Gain should be set based on echogenicity of blood in artery, ensuring that fluid remains anechoic while maximizing visibility of other structures.

^d^ Imaging Plane: Venous walls appear thin and crisp when ultrasound beam is perpendicular to walls. Avoid oblique views of veins that make walls look thickened or “fuzzy” and hinder complete compression of vein.

^e^ Compression Technique: Level of imaging should be the same after release of compression (no probe sliding). Nondiagnostic compression ultrasound exam may be due to inadequate downward compressive force (evidenced by lack of deformity of the adjacent artery), depth of the vein, or venous scarring. If the vein is truly not fully compressible, consider annotating “deep vein not fully compressible” on screen, confirming adequate compressive force was used, and the vein is not compressible.

**Table 6:** Skin & soft tissue ultrasound image quality criteria.

	Vote
**Skin and soft tissue images**	
Probe: Linear probe. Severe edema or high BMI may warrant selection of an alternative transducer offering increased depth at expense of lower resolution. For single‐transducer systems, choose linear setting.	100%
Exam Preset: Musculoskeletal or superficial	94%
Screen Orientation Marker: Upper left‐hand corner	100%
Image Orientation: In longitudinal plane, screen left is cephalad and screen right is caudad. In a transverse plane, screen left is the operator's left.^a^	100%
**Skin and subcutaneous tissue (abdominal wall)**	
Depth: Deep enough to allow visualization of subcutaneous tissue, muscle, and deep structures (bones, peritoneum, pleura, etc.).	97%
Gain: Balanced gray‐scale image in near and far fields	100%
Imaging plane: Ultrasound beam oriented either transversely or longitudinally on the skin surface. Ultrasound beam is perpendicular to skin surface to maximize resolution of subcutaneous tissue and muscles.	100%
Structures:	–
Subcutaneous tissue (fat globules) visible	100%
Muscle visible	100%
Peritoneum and loops of small bowel seen deep to the muscle when over the abdominal wall.	94%

^a^ In a transverse plane, screen left is the operator's left, which is the patient's right on the anterior body and the patient's left on the posterior body. Operators are recommended to add anatomical labels (left, right, etc.) when important for accurate interpretation.

## DISCUSSION

We have developed consensus‐based standards from a multispecialty panel of national experts to establish minimum image quality criteria for common POCUS views. These standards define the basic criteria for an adequate quality image, which has important implications for POCUS training, quality assurance, and credentialing/privileging. By establishing minimum image quality criteria for POCUS, hospitals and health systems can advance one step closer to standardization of POCUS imaging across specialties, which can ultimately improve patient safety and clinical outcomes.

Developing standard criteria to define adequate quality POCUS images is critical for POCUS training. In recent years, POCUS training has been integrated into undergraduate and graduate medical education.^31^, ^32^ For some specialties, the Accreditation Council for Graduate Medical Education (ACGME) has mandated ultrasound training as a core program requirement, including emergency medicine, critical care, and family medicine.^33^ For clinicians in practice, lack of training has been a top barrier to POCUS use in many specialties, and limited opportunities for training exist.^34^, ^35^, ^36^, ^37^, ^38^, ^39^ Regardless of level of training, gaining competence in image acquisition demands substantial effort of all novice POCUS users.

Since most institutions have relatively few POCUS experts to supervise image acquisition in real time, the development of an image portfolio with asynchronous review by experts has evolved to become a common method for demonstrating competence in image acquisition.^8^, ^9^, ^10^, ^11^, ^40^, ^41^, ^42^ However, a major challenge in reviewing POCUS image portfolios has been variability in rating the quality of images among experts. In general, when inter‐rater reliability has been assessed for various clinical findings on POCUS images, only a moderate level of agreement has been achieved (Kappa scores of 0.40–0.60).^24^, ^25^, ^26^, ^27^, ^28^, ^29^, ^30^ Historically, a lack of consensus‐based criteria to assess the quality of normal or abnormal ultrasound images has led to variability in ratings, and most published image scoring protocols lack specific image requirements to define an acceptable quality image.^19^, ^20^, ^21^, ^22^, ^23^ Some recent studies assessing inter‐rater reliability among experts have first gathered consensus on the image characteristics considered to be essential before evaluating inter‐rater reliability.^28^ Our checklist helps bridge this gap by gathering consensus across a large, multispecialty group of POCUS experts to define minimum image quality criteria for several common POCUS applications used by clinicians. Notably, our use of a modified Delphi approach allowed for open discussion among experts to ensure important nuances were incorporated to optimize the checklist.

Besides its utility in evaluating image portfolios for training, defining image quality criteria has major implications for POCUS credentialing and quality assurance.^13^, ^14^, ^40^ Variability in rating POCUS images can lead to clinicians inappropriately receiving or not receiving institutional privileges to use POCUS in patient care, and both scenarios can be potentially harmful to patients. By standardizing image quality criteria, POCUS directors and credentialing committees can systematically assess clinician skills and grant POCUS privileges using objective data. As the number of POCUS users in different specialties continues to increase exponentially, these subtleties in POCUS privileging are growing in importance. Our approach may serve as a model for defining image quality criteria for a broad range of POCUS images that are acquired by different specialties.

A limitation of our study was the exclusion of abnormal findings. We focused on the quality and structures required to define a minimum passing standard for common POCUS views. We were unable to include the potentially large number of abnormal findings and consider the image quality standards for each pathology. Such a checklist would likely be considered impracticable by most clinician experts reviewing POCUS images. For a few common conditions where abnormal findings would warrant a change in imaging technique, such as pleural effusion or lung consolidation, we included additional checklist items for the minimum image criteria. Additionally, these criteria will not apply in all clinical scenarios. For example, while chest compressions are ongoing during cardiopulmonary resuscitation, a suboptimal subcostal four‐chamber view may be adequate to rule out large pericardial effusion and cardiac tamponade and guide immediate clinical decision‐making. A strength of our study was the assembly of a multispecialty expert panel with geographic diversity from >30 different institutions. Through group discussion of items that had achieved consensus, we were able to share perspectives across specialties and capture important nuances to improve checklist items.

Validation of these consensus‐based image quality criteria in clinical practice is an important next step. By comparing the independent ratings of experts viewing the same recorded ultrasound images, we can evaluate the inter‐ and intra‐rater reliability. Additionally, qualitative research is needed to prioritize the different image quality criteria. This would allow weighing of criteria with higher importance and identifying critical flaws that would render an image uninterpretable.

These criteria shall be revisited periodically as portable ultrasound devices, especially handhelds, and imaging standards evolve with the integration of artificial intelligence in the coming years. The criteria differentiating high‐ versus low‐quality images can be integrated into artificial intelligence algorithms to standardize and automate evaluation of ultrasound image quality, streamlining the quality assurance processes for training and credentialing practices.

## CONCLUSIONS

We have defined the minimum image quality criteria for common POCUS applications by gathering consensus among a large, multispecialty group of POCUS experts. These standards address a fundamental gap in evaluating POCUS image quality and provide a foundation for consistent training, quality assurance, credentialing, and clinical practice across health systems. Our approach and findings can guide clinicians and health systems seeking to standardize and promote high‐quality POCUS imaging across specialties.

## CONFLICT OF INTEREST STATEMENT

Ria Dancel is a consultant for Fujifilm‐Sonosite. Renee Dversdal is a chief medical officer of Vave Health. The remaining authors declare no conflicts of interest.

## Supporting information

Appendix 1 ‐ Multisystem Image Checklist‐07012025.
