# Supplementary material for: Defining minimum image quality criteria for common diagnostic point‐of‐care ultrasound images: A position statement of the Society of Hospital Medicine
Source: J Hosp Med. 2025 Sep 22;21(2):183–96. doi: 10.1002/jhm.70156 (PMC12865264; doi:10.1002/jhm.70156)
Supplement: Supplementary file 1 — Appendix 1 ‐ Multisystem Image Checklist‐07012025. [file JHM-21-183-s001.docx]

**Appendix 1: Multi-system Image Quality Criteria Checklist**

| **Cardiac Images** | **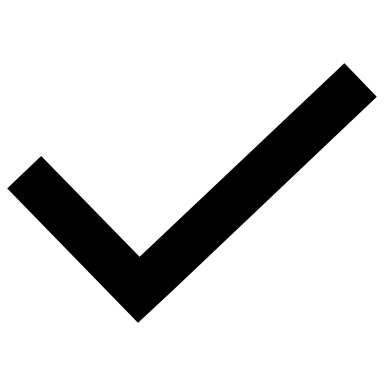** |
| --- | --- |
| **Probe:** Phased Array |  |
| **Preset:** Cardiac |  |
| **Orientation Marker**: Upper Right (*May vary based on institution and clinical specialty) |  |
| **Parasternal Long-Axis View** | **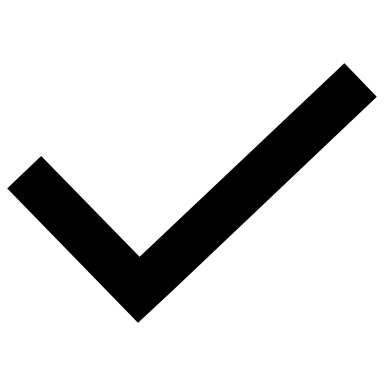** |
| **Image Orientation:** Base of the heart (AV and LA) on screen right |  |
| **Depth:** Includes descending thoracic aorta (typically 13-16 cm); No excessive depth beyond aorta. |  |
| **Gain:** Balanced gray-scale image in near and far field with blood appearing anechoic in all chambers |  |
| **Imaging plane:** Beam aligned over center of long-axis of LV with LV cavity at fullest diameter |  |
| **Structures:** |  |
| AV visible and slightly right of midline |  |
| MV visible and approximately centered in the image |  |
| RVOT is visible |  |
| LV is visible. (*Apex is generally not visible) |  |
| LA is visible. |  |
| Aortic Root is visible. |  |
| **Parasternal Short-Axis View (Aortic valve level)** | **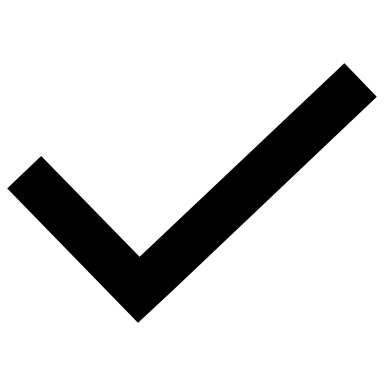** |
| **Image Orientation:** RA on screen left. RVOT on screen right. RV on top. LA on bottom. AV in center. |  |
| **Depth:** Includes inferior wall of the LA without excessive depth. |  |
| **Gain:** Balanced gray-scale image in near and far field. |  |
| **Imaging plane:** Aligned over AV with AV leaflets, RA, RV and LA seen. |  |
| **Structures:** |  |
| Right coronary cusp (CC), non-CC, left CC of the AV should be seen |  |
| LA, Interatrial Septum, RA, TV, and RV should be visible. |  |
| **Parasternal Short-Axis View (Mitral valve level)** | **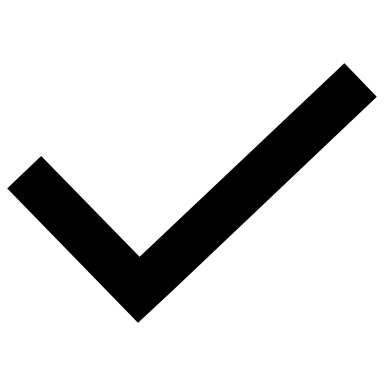** |
| **Image Orientation:** LV on screen right. RV on left or center of screen. |  |
| **Depth:** Include the inferior wall of the LV avoiding excessive depth. |  |
| **Gain:** balanced gray-scale image in near and far field |  |
| **Imaging plane:** Aligned over MV. Avoid oblique imaging planes that make the LV look oval or oblong. |  |
| **Structures:** |  |
| Anterior and posterior leaflets of the MV should be seen |  |
| Septum and RV visible (entire RV free wall may not be seen) |  |
| Endocardium of all walls (anterior, septal, inferior, lateral) of LV are visible. |  |
| **Parasternal Short-Axis View (Mid-papillary level)** | **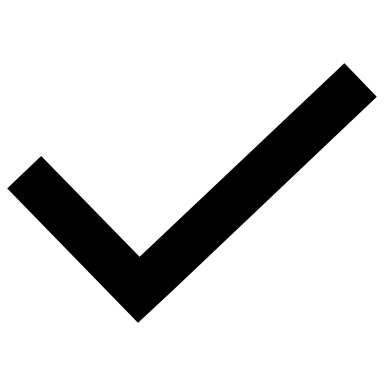** |
| **Image Orientation:** LV on screen right. RV is on left or center of screen. |  |
| **Depth:** Sufficient to include inferior wall of LV but avoiding excessive depth. |  |
| **Gain:** Balanced gray-scale image in near and far field |  |
| **Imaging plane:** Beam should be aligned over papillary muscles of LV in a short axis mid-ventricular  view. MV is not seen. Avoid oblique imaging planes that make LV look oval or oblong. |  |
| **Structures:** |  |
| Both papillary muscles are visible and symmetric |  |
| Septum and RV visible |  |
| LV endocardium of all walls visible. |  |
| **Parasternal Short-Axis View (Apical level)** | **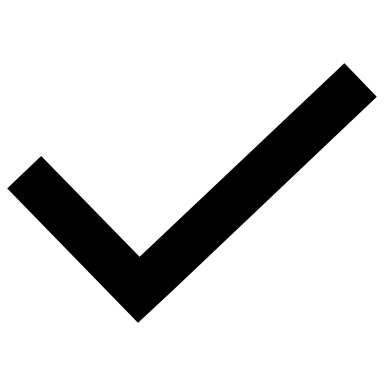** |
| **Image Orientation:** LV should be in center of screen. |  |
| **Depth:** Sufficient to include the inferior wall of the LV but avoiding excessive depth. |  |
| **Gain:** Balanced gray-scale image in near and far field |  |
| **Imaging plane:** Beam should be aligned over apex. The papillary muscles should not be seen. Avoid  oblique imaging planes that make LV look oval or oblong. |  |
| **Structures:** |  |
| LV endocardium of all walls visible. |  |
| **Apical Four-Chamber View** | **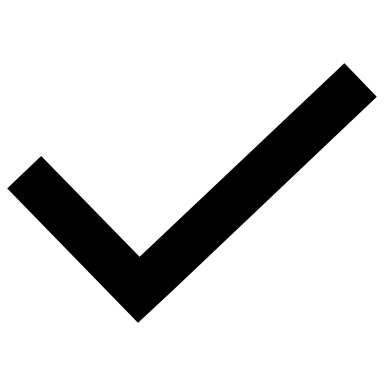** |
| **Image Orientation:** RA and RV on screen left, LA and LV on the right of the screen. |  |
| **Depth:** Sufficient to include entirety of both atria |  |
| **Gain:** Balanced gray-scale image in near and far field |  |
| **Imaging Plane:** |  |
| Apex centered on the screen with interventricular septum vertical. Ventricles should be  elongated. Normally, LV is oval and RV is triangular. |  |
| Lateral walls of both the RV and LV should be visible (entire RV free wall may not be seen). |  |
| Posterior coronary sinus should not be visible (under tilting / tilted too posteriorly). |  |
| Aortic root is not visible in an apical 4-chamber view (over tilting / tilted too anteriorly). |  |
| **Structures:** |  |
| TV well visualized including valve tips indicating RV is at greatest diameter |  |
| MV well visualized including valve tips indicating that the LV at greatest diameter |  |
| LV visible |  |
| LA visible |  |
| RV visible (entire RV free wall may not be seen). |  |
| RA visible |  |
| **Subcostal Four-Chamber View** | **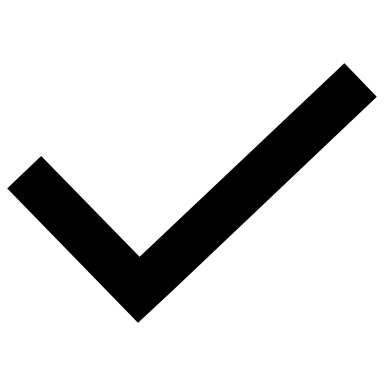** |
| **Image Orientation:** Base of heart (LA and RA) on screen left and ventricles on right of screen. |  |
| **Depth:** Sufficient to include inferolateral wall of LA in the far field |  |
| **Gain:** Balanced gray-scale image in near and far field |  |
| **Imaging plane:** |  |
| All 4 chambers should be clearly seen. |  |
| Aortic valve and aortic root not seen. |  |
| Ventricles elongated; normally, LV is oval and RV is triangular shape (apex is generally not seen) |  |
| **Structures:** |  |
| TV visible |  |
| MV visible |  |
| LV visible |  |
| RV visible |  |
| RA visible |  |
| LA visible |  |
| Liver and diaphragm visible |  |
| **Inferior Vena Cava (Longitudinal View)** | **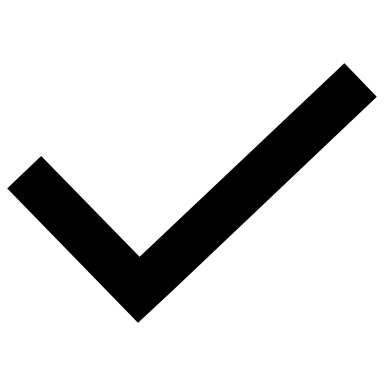** |
| **Image Orientation:** RA/diaphragm on the indicator side of screen; screen indicator cephalad |  |
| **Depth:** Deep enough to view the IVC in center of screen |  |
| **Gain:** Balanced gray-scale image in near and far field |  |
| **Imaging plane:** |  |
| Beam centered over IVC in longitudinal plane, avoiding oblique views. Anterior and posterior walls  of the IVC parallel, appearing as thin, hyperechoic lines across majority of the screen. |  |
| Should visualize IVC connecting with RA |  |
| **Structures:** |  |
| Liver and diaphragm visible |  |
| IVC in long axis is visible |  |
| Hepatic vein is visible (may not be seen in all patients) |  |

| **Lung and Pleural Images** | **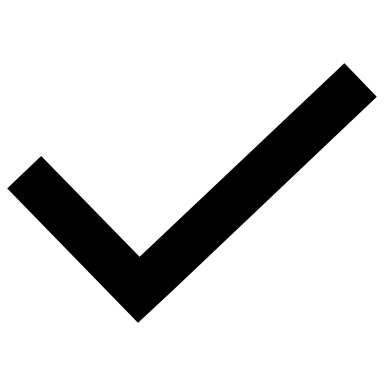** |
| --- | --- |
| **Probe:** Phased-array or curvilinear when evaluating pleura, pleural space, or parenchyma. Linear  probe when evaluating pleura only. |  |
| **Exam Preset:** Abdomen or Lung |  |
| **Orientation Marker:** Upper left-hand corner |  |
| **Image Orientation:** Screen left cephalad and screen right caudal |  |
| **Normal Lung Sliding with A-lines** | **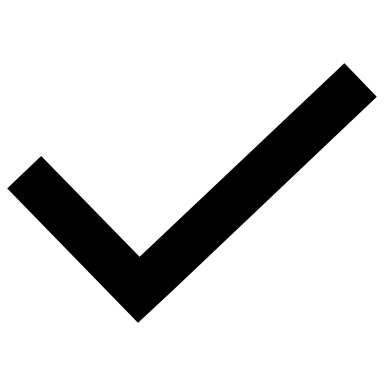** |
| **Depth:** Minimum depth typically 10-12cm, should be deep enough to see greater than 2 A-lines. |  |
| **Gain:** Balanced gray-scale image in near and far field |  |
| **Imaging plane:** Beam perpendicular to the pleural line to bring out A-lines. A thin, crisp pleural line  normally seen. Orientation cephalo-caudad with rib shadows flanking both or at least one edge of  the image. |  |
| **Structures:** |  |
| Pleural line visible |  |
| A-lines (>2 should be clearly visible) |  |
| Rib + rib shadow visible |  |
| **B-Lines** | **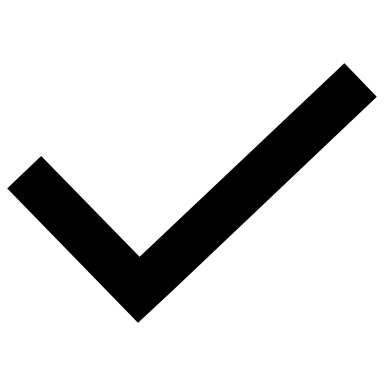** |
| **Gain:** Balanced gray-scale image in near and far field |  |
| **Imaging plane:** Beam perpendicular to pleural line. Orientation longitudinal with rib shadows  flanking both edges of the view (or at least one side/edge) |  |
| **Structures:** |  |
| Pleural line visible |  |
| B-lines clearly visible and meet the definition of B-line. |  |
| Rib + rib shadow visible |  |
| **Consolidation (or Hepatization)** | **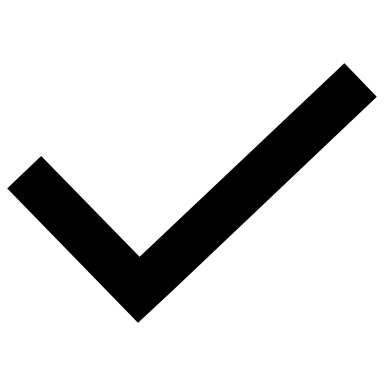** |
| **Depth:** Sufficient depth to visualize extent of consolidation or hepatization. |  |
| **Gain:** Balanced gray-scale image in near and far field |  |
| **Imaging plane:** Beam perpendicular to pleural line with rib shadows flanking both edges of view (or  at least one side/edge). Indicator side of screen must be cephalad (lung). |  |
| **Structures:** |  |
| Pleural line visible |  |
| Consolidation of lung clearly visible |  |
| Rib + rib shadow(s) visible |  |
| **Pleural Effusion** | **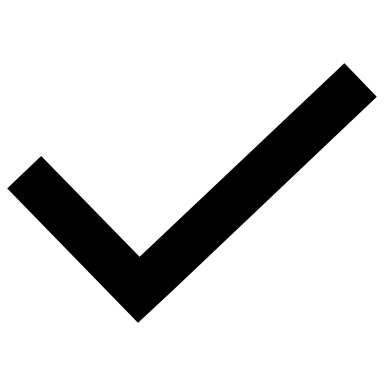** |
| **Depth:** Deep enough to see spine and visualize size of pleural effusion |  |
| **Gain:** Balanced gray-scale image in near and far field |  |
| **Imaging plane:** Beam perpendicular to chest wall with diaphragm, lung parenchyma, and pleural  effusion clearly visualized. Orientation longitudinal with lung parenchyma on the indicator side of  screen and diaphragm on opposite side. |  |
| **Structures:** |  |
| Pleural effusion visible |  |
| Lung parenchyma visible, unless pleural effusion spans multiple ribs spaces. |  |
| Diaphragm visible |  |
| Spine visible when probe lateral |  |

| **Abdominal Images** | **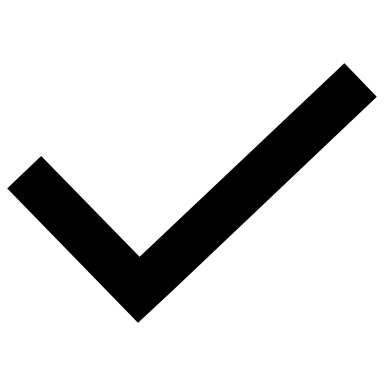** |
| --- | --- |
| **Probe:** Curvilinear or phased-array probe |  |
| **Exam Preset:** Abdomen (or specific preset for FAST, Aorta, Liver, etc.) |  |
| **Screen Orientation Marker:** Upper left-hand corner |  |
| **Image Orientation:** In a longitudinal plane, screen left is cephalad and screen right is caudal. In a transverse plane, screen left should be patient’s right and screen right is patient’s left. |  |
| **Right Kidney with Hepatorenal Recess (Longitudinal View)** | **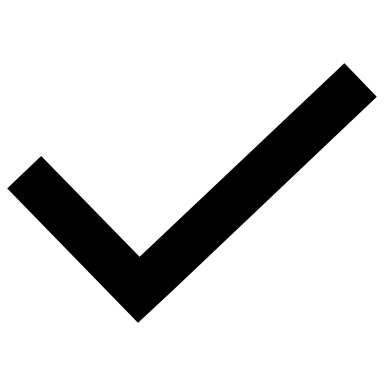** |
| **Depth:** Includes entire kidney in center of the screen |  |
| **Gain:** Balanced gray-scale image in near and far field |  |
| **Imaging plane:** Beam aligned longitudinally over hilum of the kidney with both superior and inferior  poles visualized by rocking or sliding during image recording. Kidney should appear oval shaped  and not circular. Maximize kidney length with probe oriented along the rib spaces, minimizing rib  shadows. |  |
| **Structures:** |  |
| Renal cortex and pelvis visible (Typically, renal vessels are visualized in the pelvis but not required) |  |
| Superior and inferior poles of kidney visible (If both poles cannot be seen simultaneously, rock or  slide probe |  |
| Liver and part of hepatorenal recess visible |  |
| **Right Kidney (Transverse View)** | **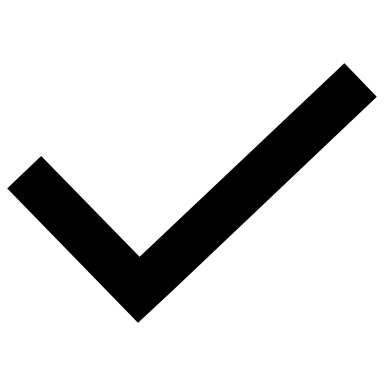** |
| **Depth:** Deep enough to center kidney on the screen. |  |
| **Gain:** Balanced gray-scale image in near and far field. |  |
| **Imaging plane:** Beam aligned transversely through the center of the kidney with hilar vessels and  collecting ducts visible. |  |
| Superior and inferior poles of kidney visualized by tilting or fanning probe. |  |
| **Left Kidney with Splenorenal Recess (Longitudinal view)** | **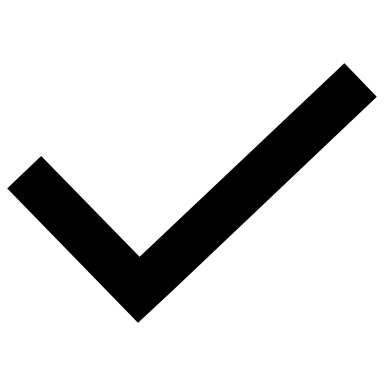** |
| **Depth:** Deep enough to include entire kidney in center of screen |  |
| **Gain:** Balanced gray-scale image in near and far field |  |
| **Imaging plane:** Beam aligned longitudinally over hilum of the kidney with both superior and inferior  poles visualized by rocking or sliding during image recording. Kidney should appear oval shaped  and not circular. Maximize kidney length with probe oriented along the rib spaces, minimizing rib  shadows |  |
| **Structures:** |  |
| Renal cortex and pelvis visible (Typically, renal vessels visualized in the pelvis but not required) |  |
| Superior and inferior poles of kidney (If both poles cannot be seen simultaneously, rock or slide  Probe) |  |
| Spleen and part of splenorenal recess visible |  |
| **Left Kidney (Transverse View)** | **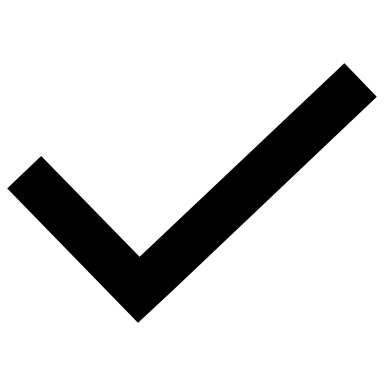** |
| **Depth:** Deep enough to center kidney on the screen. |  |
| **Gain:** Balanced gray-scale image in near and far field. |  |
| **Imaging plane:** Beam aligned transversely through center of the kidney with hilar vessels and  collecting ducts visible. |  |
| Superior and inferior poles of kidney visualized by tilting or fanning probe. |  |
| **Abdominal Aorta (Transverse View)** | **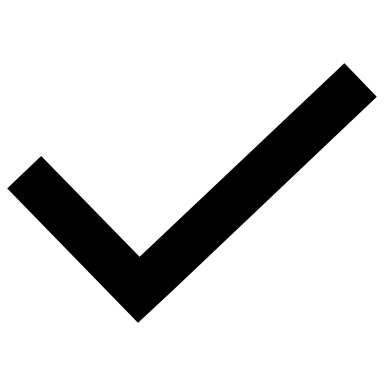** |
| **Depth:** Sufficient to visualize vertebral shadow |  |
| **Gain:** Balanced gray-scale image in near and far field |  |
| **Imaging plane:** Beam aligned transversely and perpendicular to the walls of the aorta. The anterior  and posterior walls of the circular aorta seen approximately in center of screen. |  |
| **Structures:** |  |
| Proximal Aorta with celiac trunk or superior mesenteric artery, IVC, and vertebral shadow visible.  Diameter measured at the widest point at Proximal, Mid, and Distal aorta, either outer wall to  outer wall or from leading edge to leading edge based on institutional protocol. |  |
| Mid aorta with IVC and vertebral shadow visible. |  |
| Distal aorta w/ transition to the common iliac arteries with vertebral shadow visible. |  |
| **Abdominal Aorta (Longitudinal View)** | **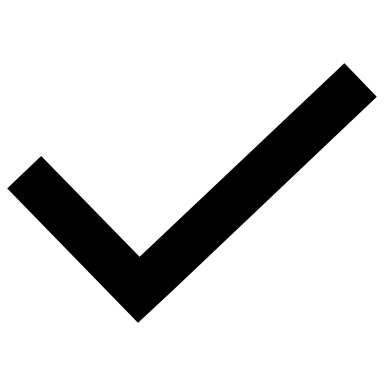** |
| **Depth:** Sufficient to visualize vertebral shadow |  |
| **Gain:** Balanced gray-scale image in near and far field |  |
| **Imaging plane:** Beam aligned longitudinally over the center of aorta without any narrowing or  cutting off the aorta. Two walls of the aorta seen longitudinally across screen from left to right.  Beak-shaped, oblique view of aorta avoided. |  |
| **Structures:** |  |
| Proximal, mid, or distal abdominal aorta in long axis visible |  |
| Spine visible |  |
| Two branches of aorta (celiac trunk, superior mesenteric artery) visible |  |
| **Urinary Bladder (Transverse View)** | **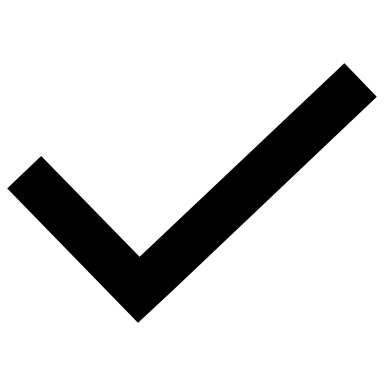** |
| **Depth:** Deep enough to see entire posterior wall of bladder. Depth should be adequate to see pelvic  organs (rectum, prostate, or uterus if present) |  |
| **Gain:** Balanced gray-scale image in near and far field (Adjust far-field gain to minimize posterior  acoustic enhancement |  |
| **Imaging plane:** Beam centered transversely over bladder in center of screen. Images should capture  maximum dimensions of bladder for proper assessment of volume. |  |
| **Structures:** |  |
| Bladder in a transverse plane visible |  |
| Pelvic organs (rectum, and prostate or uterus if present) must be seen in far field |  |
| **Urinary Bladder (Longitudinal View)** | **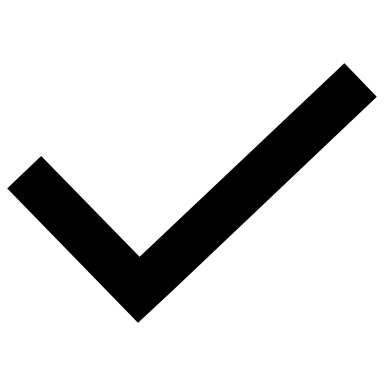** |
| **Depth:** Deep enough to see posterior wall of the bladder, rectum, prostate, or uterus if present. |  |
| **Gain:** Balanced gray-scale image in near and far field |  |
| **Imaging plane:** Beam centered longitudinally over bladder in center of screen. Images should  capture maximum dimensions of the bladder for proper assessment of volume. Typically, probe is  positioned over superior posterior edge of the symphysis pubis and the rocked with ultrasound  beam aimed toward the pelvis in order to capture a high-quality view. |  |
| **Structures:** |  |
| Bladder in long axis visible |  |
| Pelvic organs (rectum, prostate and uterus if present) are visible in far field |  |
| Symphysis pubis + shadow (screen right) visible (Shadow may not be seen if bladder is greatly  distended |  |

| **Lower Extremity Deep Venous Thrombosis Exam Images** | **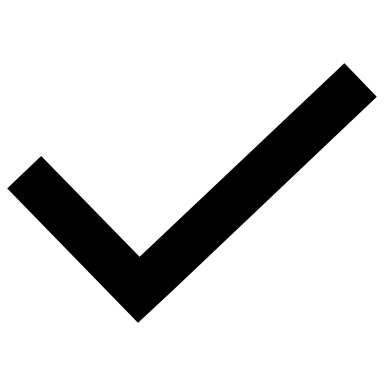** |
| --- | --- |
| **Probe:** Linear probe. Severe edema or high BMI may warrant selection of an alternative transducer  offering increased depth at expense of lower resolution. For single-transducer systems, choose  the linear setting. |  |
| **Exam Preset:** Vascular, venous, arterial, or musculoskeletal |  |
| **Screen Orientation Marker:** Upper left-hand corner |  |
| **Image Orientation on Screen:** Screen Left is patient’s right, Screen Right is patient’s left with vessels centered on screen, avoiding excessive depth placing the vessels in the upper third of screen. |  |
| **Right Common Femoral Vein** | **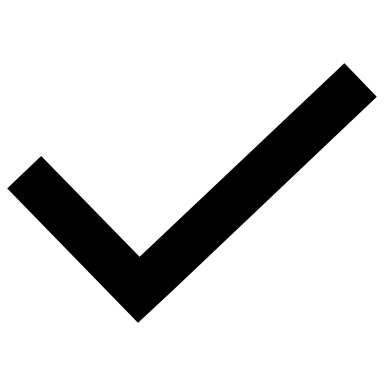** |
| **Depth:** Sufficient to visualize the right CFV in center of screen |  |
| **Gain:** Balanced gray-scale image in near and far field |  |
| **Imaging plane:** Ultrasound beam should be transverse to target vessel and vein should appear in  center of screen (avoid oblique views) |  |
| **Structures:** |  |
| Both right CFA (screen left) and right CFV (screen right) are visible |  |
| GSV should not be visible |  |
| Full compressibility of right CFV demonstrated without sliding. |  |
| **Right Common Femoral Vein - Greater Saphenous Vein Junction** | **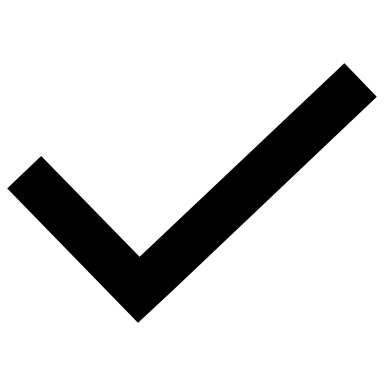** |
| **Depth:** Sufficient to visualize CFV-GSV in the center of the screen |  |
| **Gain:** Balanced gray-scale image in near and far field |  |
| **Imaging plane:** Ultrasound beam should be transverse to target vessel and vein should appear in  center of screen (avoid oblique views) |  |
| **Structures:** |  |
| Right CFA, CFV and GSV all visible, with GSV extending medially and anteriorly (top right for RLE) |  |
| Full compressibility of both right CFV and GSV demonstrated without sliding. |  |
| **Right Femoral Vein - Deep Femoral Vein Junction** | **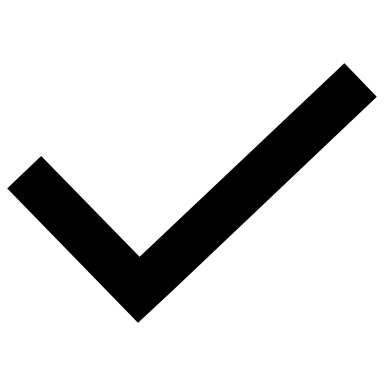** |
| **Depth:** Sufficient to visualize the right CFV splitting into FV and DFV branches in center of screen.  Note: lateral perforators are usually seen between superficial and deep femoral artery and should  NOT be confused with DFV. After CFV splits into FV and DFV, DFV usually dives posteriorly and is  not seen distally beyond bifurcation. |  |
| **Gain:** Balanced gray-scale image in near and far field |  |
| **Imaging plane:** Ultrasound beam should be transverse to target vessel and vein should appear in  center of screen (avoid oblique views) |  |
| **Structures:** |  |
| Distal right CFV and bifurcation both visible with slight tilting or fanning of the probe |  |
| Right FV and DFV visible (avoid mistaking lateral perforator vein for DFV) |  |
| Full compressibility of right FV and DFV, and any lateral perforators, demonstrated without sliding. |  |
| **Right Mid-Distal Femoral Vein** | **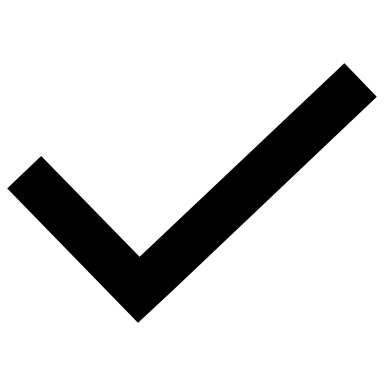** |
| **Depth:** Sufficient to visualize the right mid-/distal FV in center of screen |  |
| **Gain:** Balanced gray-scale image in near and far field |  |
| **Imaging plane:** Ultrasound beam should be transverse to target vessel and vein should appear in  center of screen (avoid oblique views) |  |
| **Structures:** |  |
| Right Mid-/distal FV and superficial femoral artery visible |  |
| Full compressibility of the right FV demonstrated without sliding. To improve the sensitivity of the  DVT exam, compressions in ~1 cm increments down the length of the FV is recommended. |  |
| **Right Popliteal Vein** | **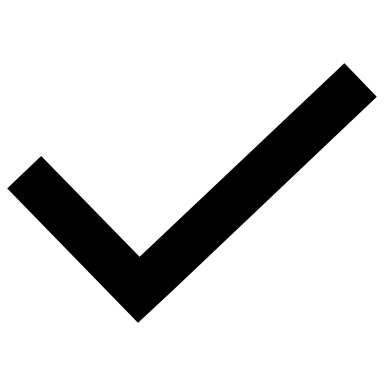** |
| **Depth:** Sufficient to visualize right PV in center of screen and not mistaken for superficial calf veins |  |
| **Gain:** Balanced gray-scale image in near and far field |  |
| **Imaging plane:** Ultrasound beam should be transverse to target vessel and vein should appear in  center of screen (avoid oblique views). Avoid capturing views too distal where the PV has bi- or  trifurcated. |  |
| **Structures:** |  |
| Right PV and PA visible |  |
| Full compressibility of the right PV should be demonstrated without sliding. Avoid mistaking the  anterior tibial, peroneal, or posterior tibial veins for the PV. |  |
| **Left Common Femoral Vein** | **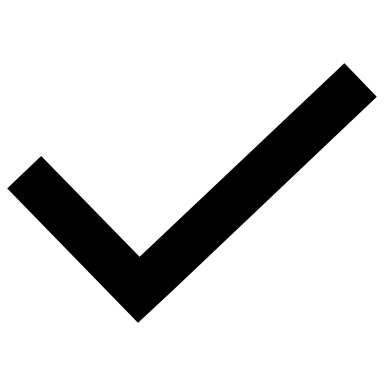** |
| **Depth:** Sufficient to visualize the left CFV in center of screen |  |
| **Gain:** Balanced gray-scale image in near and far field |  |
| **Imaging plane:** Ultrasound beam should be transverse to target vessel and vein should appear in  center of screen (avoid oblique views) |  |
| **Structures:** |  |
| Both left CFA (screen right) and left CFV (screen left) are visible |  |
| GSV should not be visible |  |
| Full compressibility of left CFV demonstrated without sliding. |  |
| **Left Common Femoral Vein - Greater Saphenous Vein Junction** | **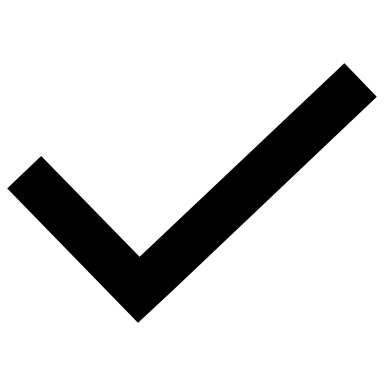** |
| **Depth:** Sufficient to visualize CFV-GSV in the center of the screen |  |
| **Gain:** Balanced gray-scale image in near and far field |  |
| **Imaging plane:** Ultrasound beam should be transverse to target vessel and vein should appear in  center of screen (avoid oblique views) |  |
| **Structures:** |  |
| Left CFA, CFV and GSV all visible, with GSV extending medially and anteriorly (top left for LLE) |  |
| Full compressibility of both left CFV and GSV demonstrated without sliding. |  |
| **Left Femoral Vein - Deep Femoral Vein Junction** | **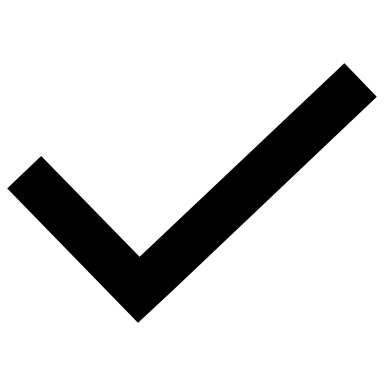** |
| **Depth:** Sufficient to visualize the left CFV splitting into FV and DFV branches in center of screen. Note:  lateral perforators are usually seen between superficial and deep femoral artery and should NOT  be confused with DFV. After CFV splits into FV and DFV, DFV usually dives posteriorly and is not  seen distally beyond bifurcation. |  |
| **Gain:** Balanced gray-scale image in near and far field |  |
| **Imaging plane:** Ultrasound beam should be transverse to target vessel and vein should appear in  center of screen (avoid oblique views) |  |
| **Structures:** |  |
| Distal left CFV and bifurcation both visible with slight tilting or fanning of the probe |  |
| Left FV and DFV visible (avoid mistaking lateral perforator vein for DFV) |  |
| Full compressibility of left FV and DFV, and any lateral perforators, demonstrated without sliding. |  |
| **Left Mid-Distal Femoral Vein** | **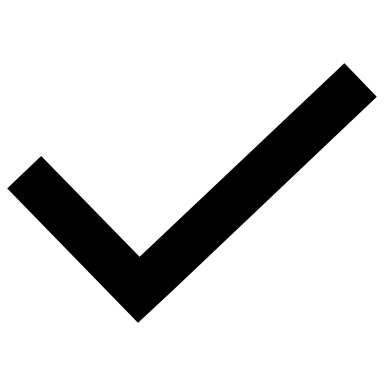** |
| **Depth:** Sufficient to visualize the left mid-/distal FV in center of screen |  |
| **Gain:** Balanced gray-scale image in near and far field |  |
| **Imaging plane:** Ultrasound beam should be transverse to target vessel and vein should appear in  center of screen (avoid oblique views) |  |
| **Structures:** |  |
| Left Mid-/distal FV and superficial femoral artery visible |  |
| Full compressibility of the left FV demonstrated without sliding. To improve the sensitivity of the  DVT exam, compressions in ~1 cm increments down the length of the FV is recommended. |  |
| **Left Popliteal Vein** | **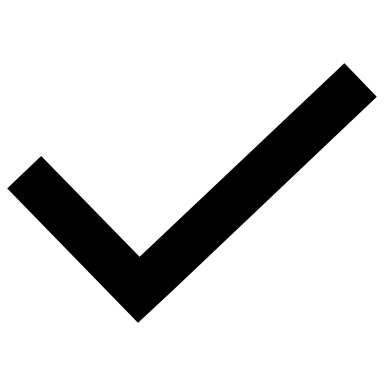** |
| **Depth:** Sufficient to visualize left PV in the center of screen and not mistaken for superficial calf veins |  |
| **Gain:** Balanced gray-scale image in near and far field |  |
| **Imaging plane:** Ultrasound beam should be transverse to target vessel and vein should appear in  center of screen (avoid oblique views). Avoid capturing views too distal where the PV has bi- or  trifurcated. |  |
| **Structures:** |  |
| Left PV and PA visible |  |
| Full compressibility of the left PV should be demonstrated without sliding. Avoid mistaking the  anterior tibial, peroneal, or posterior tibial veins for the PV. |  |

| **Skin and Soft Tissue Images** | **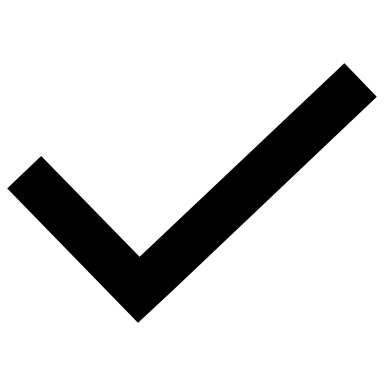** |
| --- | --- |
| **Probe:** Linear probe. |  |
| **Exam Preset:** Musculoskeletal or superficial |  |
| **Screen Orientation Marker:** Upper left-hand corner |  |
| **Image Orientation on Screen:** In a longitudinal plane, screen left is cephalad and screen right is caudal. In a transverse plane, screen left should be patient’s right and screen right is patient’s left. |  |
| **Skin and Subcutaneous Tissue (Abdominal Wall)** | **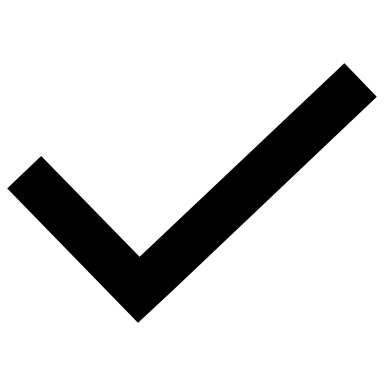** |
| **Depth:** Deep enough to allow visualization of subcutaneous tissue, muscle, and deep structures  (bones, peritoneum, pleura, etc.). |  |
| **Gain:** Balanced gray-scale image in near and far field |  |
| **Imaging plane:** Ultrasound beam oriented either transversely or longitudinally on the skin surface.  Ultrasound beam is perpendicular to skin surface to maximize resolution of subcutaneous tissue  and muscles. |  |
| **Structures:** |  |
| Subcutaneous tissue (fat globules) visible |  |
| Muscle visible |  |
| Peritoneum and loops of small bowel seen deep to the muscle when over the abdominal wall. |  |
